# Effects of copy number variations on longevity in late-onset Alzheimer’s disease patients: insights from a causality network analysis

**DOI:** 10.3389/fnagi.2023.1241412

**Published:** 2023-11-02

**Authors:** Yanan Hao, Chuhao Li, He Wang, Chen Ming

**Affiliations:** ^1^Department of Public Health and Medicinal Administration, Faculty of Health Sciences, University of Macau, Macau, Macao SAR, China; ^2^Department of Biomedical Sciences, Faculty of Health Sciences, University of Macau, Macau, Macao SAR, China; ^3^Institute of Science and Technology for Brain-Inspired Intelligence, Fudan University, Shanghai, China

**Keywords:** copy number variation, longevity, Alzheimer’s disease, causality network, plasminogen activation

## Abstract

Alzheimer’s disease (AD), particularly late-onset Alzheimer’s disease (LOAD), is a prevalent form of dementia that significantly affects patients’ cognitive and behavioral capacities and longevity. Although approximately 70 genetic risk factors linked with AD have been identified, their influence on patient longevity remains unclear. Further, recent studies have associated copy number variations (CNVs) with the longevity of healthy individuals and immune-related pathways in AD patients. This study aims to investigate the role of CNVs on the longevity of AD patients by integrating the Whole Genome Sequencing (WGS) and transcriptomics data from the Religious Orders Study/Memory and Aging Project (ROSMAP) cohort through causality network inference. Our comprehensive analysis led to the construction of a CNV-Gene-Age of Death (AOD) causality network. We successfully identified three key CNVs (DEL5006, mCNV14192, and DUP42180) and seven AD-longevity causal genes (*PLGRKT*, *TLR1*, *PLAU*, *CALB2*, *SYTL2*, *OTOF*, and *NT5DC1*) impacting AD patient longevity, independent of disease severity. This outcome emphasizes the potential role of plasminogen activation and chemotaxis in longevity. We propose several hypotheses regarding the role of identified CNVs and the plasminogen system on patient longevity. However, experimental validation is required to further corroborate these findings and uncover precise mechanisms. Despite these limitations, our study offers promising insights into the genetic influence on AD patient longevity and contributes to paving the way for potential therapeutic interventions.

## 1. Introduction

Alzheimer’s disease (AD), the most prevalent form of dementia, is estimated to affect approximately 40 million individuals globally based on estimations from WHO [Alzheimer’s Disease International (ADI), 2022; Reitz et al., 2023]. The late-onset Alzheimer’s disease (LOAD), diagnosed typically after the age of 65, accounts for about 95% of all AD cases (Bertram and Tanzi, 2005; Bekris et al., 2010). Characterized by beta-amyloid plaque and neurofibrillary tangles (DeTure and Dickson, 2019), LOAD represents a profound impact on not only cognitive and behavioral capabilities but also patient longevity. After clinical diagnosis, the average life expectancy of AD patients ranges from 4 to 8 years, though some cases report living up to 20 years with the disease (Alzheimer’s Association, 2022). Notably, a significant variation in life expectancy exists among AD patients, and the underlying genetic mechanisms contributing to this variability remain unknown.

Large-scale cohort studies focusing on Alzheimer’s disease have identified approximately 70 genetic risk factors linked with AD (Lambert et al., 2013; Marioni et al., 2018; Jansen et al., 2019; Kunkle et al., 2019; Wightman et al., 2021; Bellenguez et al., 2022; Reitz et al., 2023). Although these factors may contribute to the predisposition to AD, their impact on the longevity of patients remains largely unexplored. A previous study based on 2,872 Danish twins has observed that genetic factors can explain 25% variation in the human lifespan (Herskind et al., 1996). And several previous research has shed light on the mechanisms by which genes contribute to longevity (Conneely et al., 2012; Melzer et al., 2020). Meanwhile, several Danish and U.S. population-based cohort studies have found that genome-wide copy number variation (CNV) burden is associated with human longevity (Kuningas et al., 2011; Nygaard et al., 2016). A Han Chinese population-based GWAS study also discovered that CNVs are associated with human longevity through various aging-related phenotypes, such as telomere length, the risk of cancer, and vascular and immune-related diseases (Zhao et al., 2018). Intriguingly, our previous study revealed an association between CNVs and immune-related pathways in AD patients through WGS and transcriptomics data integration (Ming et al., 2022).

**Figure 1:**
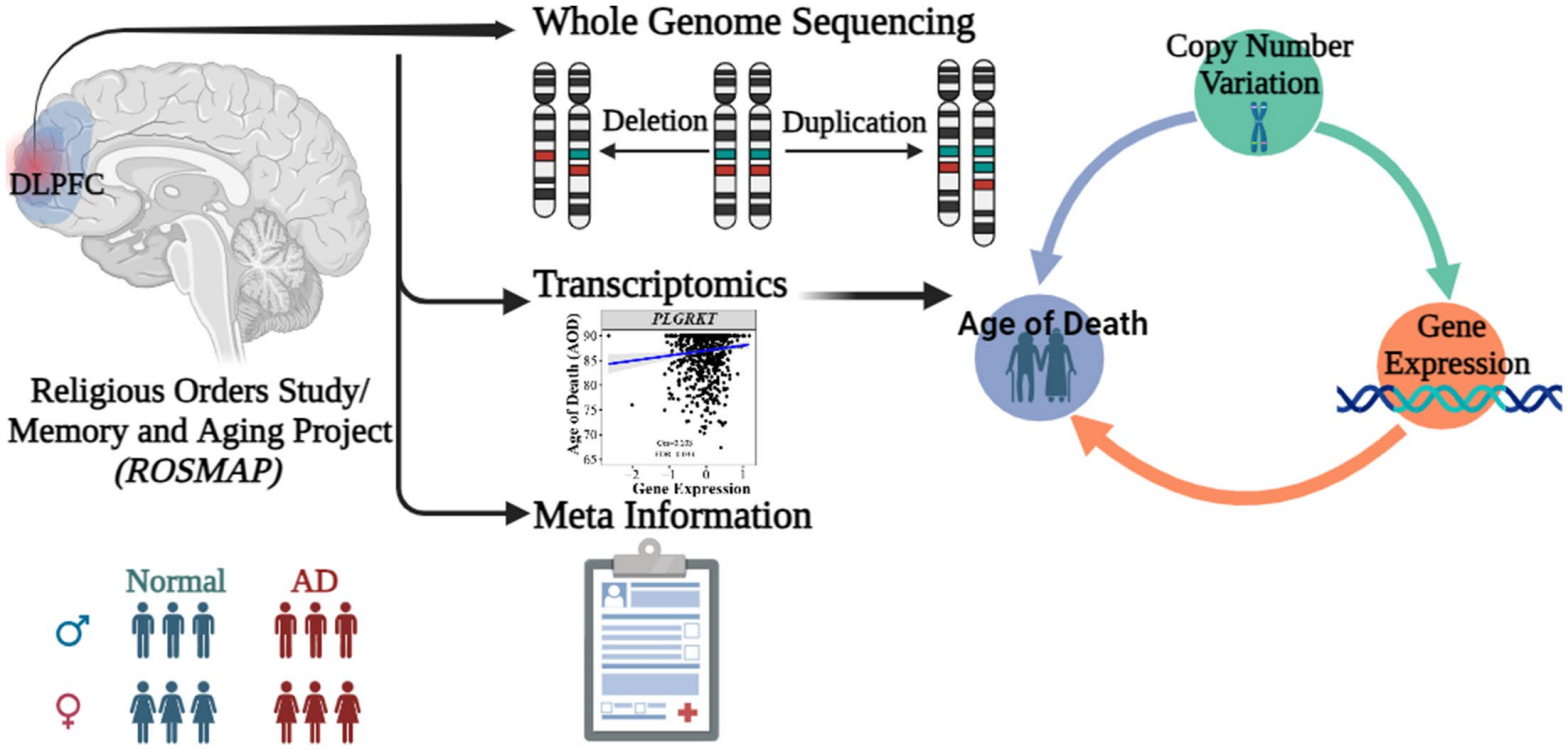
Abstract graph of integrating WGS-based CNV, transcriptomics data and meta information of ROSMAP cohort.

Motivated to unravel the influence of CNVs on the longevity of AD patients, we conducted a comprehensive analysis integrating WGS and transcriptomics data from the Religious Orders Study/Memory and Aging Project (ROSMAP) cohort (Chibnik et al., 2018; De Jager et al., 2018; Mostafavi et al., 2018; Lee et al., 2023) (Figure 1). Our research focuses on elucidating the causality network connecting copy number variations, gene expression, and the age of death in Alzheimer’s patients. Through our comprehensive analysis, we identified three key CNVs (i.e., DEL5006, mCNV14192, and DUP42180) that regulate the expression of seven genes (i.e., *PLGRKT*, *TLR1*, *PLAU*, *CALB2*, *SYTL2*, *OTOF*, and *NT5DC1*) ultimately impacting the age of death of AD patients. The AOD-correlated genes further showed functional enrichment on the plasminogen activation and chemotaxis pathway. This research sheds light on the genetic factors contributing to the discrepancy in longevity observed among AD patients and provides insights into potential therapeutic targets for improving the longevity of AD patients.

## 2. Results

### 2.1. Copy number dosages of ten CNVs are significantly correlated with the age of death of AD cases

**Figure 2:**
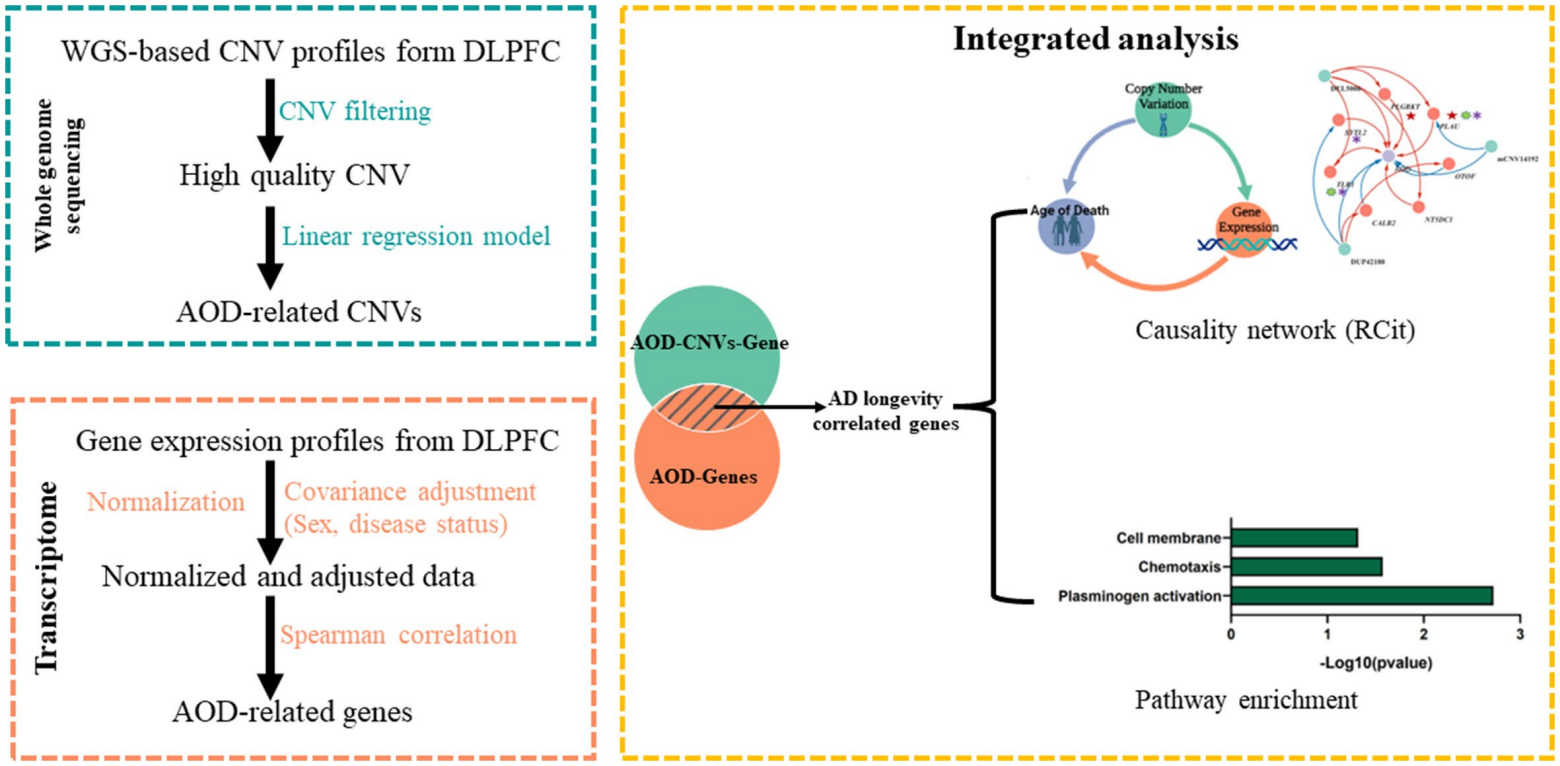
The pipeline to integrate WGS-based CNV and transcriptomics data. Green block, WGS-based CNV data; Orange block, transcriptomics data; Yellow block, integrated analysis for CNV and gene.

**Figure 3:**
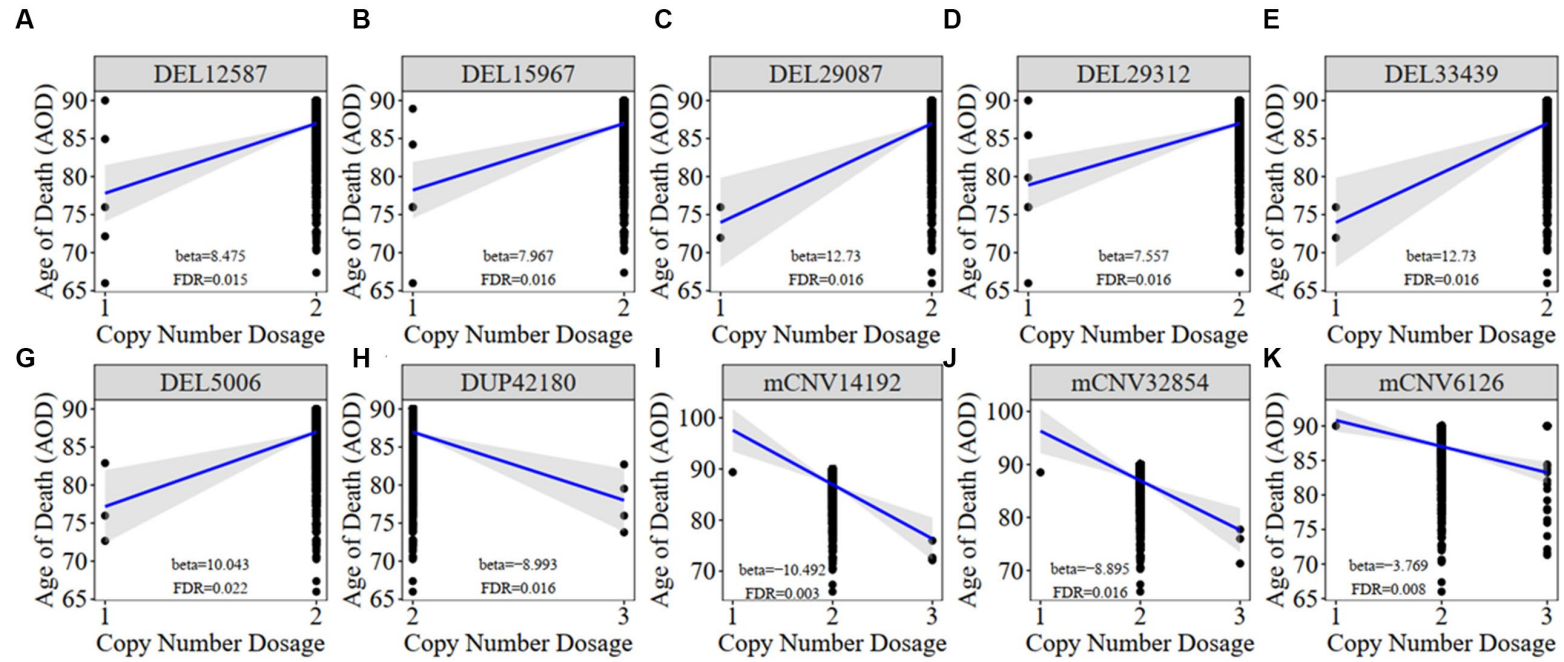
The correlation between AOD-correlated CNV and AOD. We utilized a generalized linear model in Matrix eQTL to perform correlation analysis between AOD-correlated CNV dosage and gene expression. t-statistic, False Discovery Rate (FDR) < 0.05; Beta: estimated effect size of CNV on AOD; Blue line: regression line added by R function ggscatter; Grey: the 95% confidence interval.

We constructed a linear regression model to investigate the correlation between CNVs and the age of death (AOD) of AD cases based on copy number profiles and meta-information of 1,127 North American white individuals from the ROSMAP AD cohort (Chibnik et al., 2018; De Jager et al., 2018; Mostafavi et al., 2018; Lee et al., 2023) (Supplementary Table S1 and Supplementary Figure S1), while sex and disease status were treated as covariates (Methods 4.5, Figure 2). There are 762 AD patients and 365 healthy normal (NL) individuals based on the physician’s overall cognitive diagnostic category at the time of death (cogdx) in the ROSMAP cohort. To exclude the bias from the severity of AD status, we treated various AD pathological traits, which are cogdx, Braak stage score (braaksc), and consortium to establish a registry for Alzheimer’s disease score (ceradsc), to represent the severity of AD independently in the linear regression analysis (Methods 4.5). Upon accounting for potential biases linked to sex and disease status, our analysis revealed 12, 12, and 11 AOD-correlated CNVs when incorporating cogdx, braaksc, and ceradsc as covariates, respectively. These findings each maintained a false discovery rate (FDR) of less than 0.05 (Supplementary Figure S2). There were 10 consensus AOD-correlated CNVs under all three AD pathological criteria in total (Table 1 and Figure 3). Among them, six CNVs were positively correlated with AOD, while four CNVs were negatively correlated with AOD (Table 1 and Figure 3).

**Table 1:** Summary of the significant AOD-correlated CNVs.

CNV	Chr	Start	End	Beta (CNV-AOD)	P.adj (CNV-AOD)	Total allele count (AC)	Deletion AC	Duplication AC	Allele number (AN)	Allele frequency (AF)	CNV carrier frequency (ROSMAP)	Location annotation
DEL33439	chr11	28,670,388	28,698,217	1.27E+01	1.58E-02	2	2	-	2,254	0.0887%	0.18%	Intron (NM_022763, intron 3 of 25)
DEL12587	chr3	103,265,515	103,266,967	8.48E+00	1.47E-02	5	5	-	2,254	0.2218%	0.44%	Promoter-TSS (NM_001042454)
mCNV32854	chr10	125,318,955	125,323,070	−8.89E+00	1.58E-02	4	1	3	2,254	0.1775%	0.35%	Intergenic
DEL29312	chr9	24,099,045	24,104,914	7.56E+00	1.58E-02	6	6	-	2,254	0.2662%	0.53%	Intron (NM_001130987, intron 52 of 55)
mCNV14192	chr3	171,925,368	171,927,349	−1.05E+01	3.25E-03	4	1	3	2,254	0.1775%	0.35%	Intergenic
DUP42180	chr16	31,476,591	31,489,411	−8.99E+00	1.58E-02	4	-	4	2,254	0.1775%	0.35%	Intergenic
DEL5006	chr2	17,194,382	17,196,437	1.00E+01	2.19E-02	3	3	-	2,254	0.1331%	0.27%	Intergenic
mCNV6126	chr2	71,904,772	71,907,076	−3.77E+00	7.97E-03	28	1	27	2,254	1.2422%	2.48%	Intergenic
DEL29087	chr9	11,470,585	11,635,259	1.27E+01	1.58E-02	2	2	-	2,254	0.0887%	0.18%	Intergenic
DEL15967	chr4	64,478,325	64,483,686	7.97E+00	1.58E-02	5	5	-	2,254	0.2218%	0.44%	Intergenic

The genomic coordinates are based on HG19 reference genome. Beta, estimated effect size; P.adj, adjusted *p*-value calculated based on linear regression by Matrix eQTL and adjusted by FDR; AC, the chromosome-level allele count; AN, total chromosome number; AF, the chromosome-level allele frequency; CNV carrier frequency, the frequency of DEL/DUP carriers in the ROSMAP cohort.

Meanwhile, we classified all individuals into different groups based on the copy number dosage of each AOD-correlated CNV and applied survival analysis (Method 4.12). Copy number dosage had a significant influence on AOD and that was consistent with our correlation result between these 10 CNVs and AOD, except mCNV62126 (Supplementary Figure S3). Individuals with either deletion or duplication of AOD-correlated CNVs exhibited different lifespan compared to individuals with copy number dosage of two.

To investigate whether there are significant differences in frequency between the AD and healthy control groups for the above 10 AOD-CNVs, we compared their frequency using the chi-square test (Methods 4.6). We found all 10 AOD-correlated CNVs with low frequency in both AD and healthy control groups, and they showed no significant frequency difference in both groups (Supplementary Table S2). To further investigate the prevalence of the above AOD-correlated CNVs in the human population, we compared our AOD-correlated CNVs with four public CNV datasets based on large populations, i.e., GnomAD (Chen et al., 2022), DECIPHER (Firth et al., 2009), Database of Genomic Variants (DGV) (MacDonald et al., 2014) and the 1,000 Genome Project (Sudmant et al., 2015). Nine CNVs were validated in the public CNV databases with more than 50% reciprocal overlapping ratio and with same CNV type. They also showed rare frequency in human populations (Supplementary Table S2).

### 2.2. The AOD-correlated CNVs regulate the expression of seventeen AOD-correlated genes in the DLPFC region of AD cases

To further explore how AOD-correlated CNVs regulate gene expression in the brains of AD cases, we implemented the expression quantitative trait loci (eQTL) analysis by using Matrix eQTL software (Shabalin, 2012) to integrate genome-wide CNV profiles and transcriptomics data of the dorsal lateral prefrontal cortex (DLPFC) region from the ROSMAP cohort (Methods 4.7, Figure 2). Based on the linear additive model with sex and disease status as covariates, there were 110 genes identified with their expression level significantly correlated with copy number dosage of the 10 AOD-correlated CNVs under the FDR threshold at 5%, forming 125 CNV-Gene pairs (Supplementary Table S3).

To pinpoint AOD-correlated genes, we further performed Spearman correlation analysis between all genes identified in the transcriptomics data of the DLPFC region and AOD (Methods 4.8). The transcriptomic profile was further adjusted for AD pathological traits using limma R package (Ritchie et al., 2015) to exclude bias from disease status (Supplementary Figure S4, Methods 4.8). There were 2,056, 1,294, and 2,774 AOD-correlated genes identified based on cogdx, braaksc, and ceradsc respectively, under the genome-wide FDR threshold of 0.05 (Supplementary Table S4).

**Figure 4:**
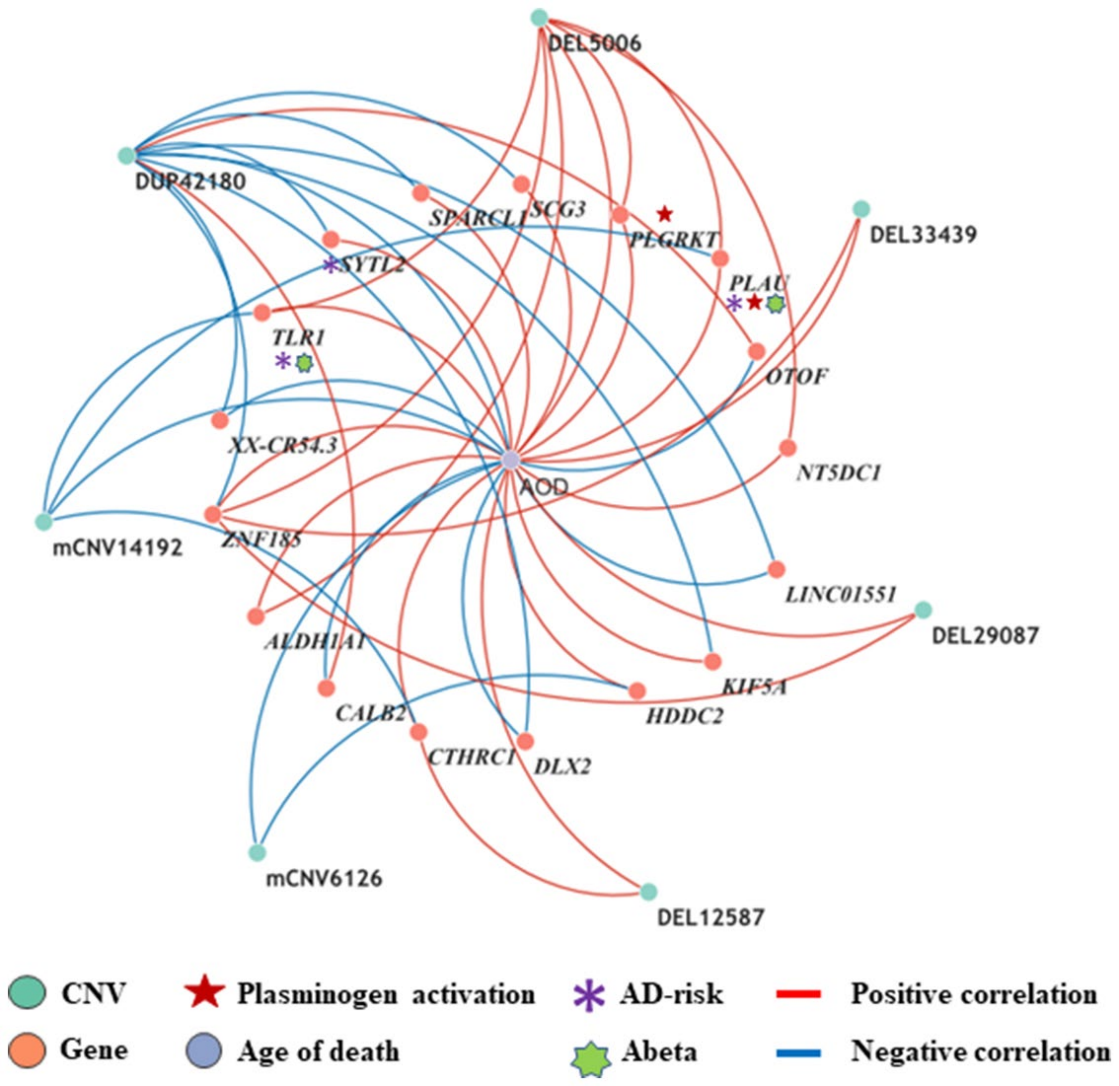
The correlation network between AOD-correlated CNVs, Gene expression, and AOD. The correlation network connected 9 AOD-correlated CNVs, 17 AOD-correlated genes, and age of death (AOD). Tangerine pink: genes, Turquoise blue: AOD-correlated CNVs; Lavender purple: AOD; Red lines: positive correlation; Blue lines: negative correlation; Red five-pointed star: plasminogen activation-related genes; Green seven-pointed star, Abeta genes; Purple asterisk, AD-risk genes.

By intersecting the 2,056, 1,294 and 2,274 AOD-correlated genes with the 110 genes in the 125 CNV-Gene pairs, we finally pinpointed 17, 14, 21 genes (based on cogdx, braaksc, and ceradsc respectively) which were both correlated with AOD and AOD-correlated CNVs respectively, defined as presumptive AD longevity-correlated genes. We used 17 genes underlying cogdx for further analysis and these genes formed 23 CNV-Gene-AOD pairs with 9 AOD-correlated CNVs. We further constructed correlation network for above CNV-gene-AOD pairs. (Figure 4; Supplementary Table S5; Methods 4.9).

### 2.3. Causality network between CNVs, gene expression, and age of death of AD patients pinpointed three key CNVs, seven key genes and showed enrichment on plasminogen activation pathway

**Figure 5:**
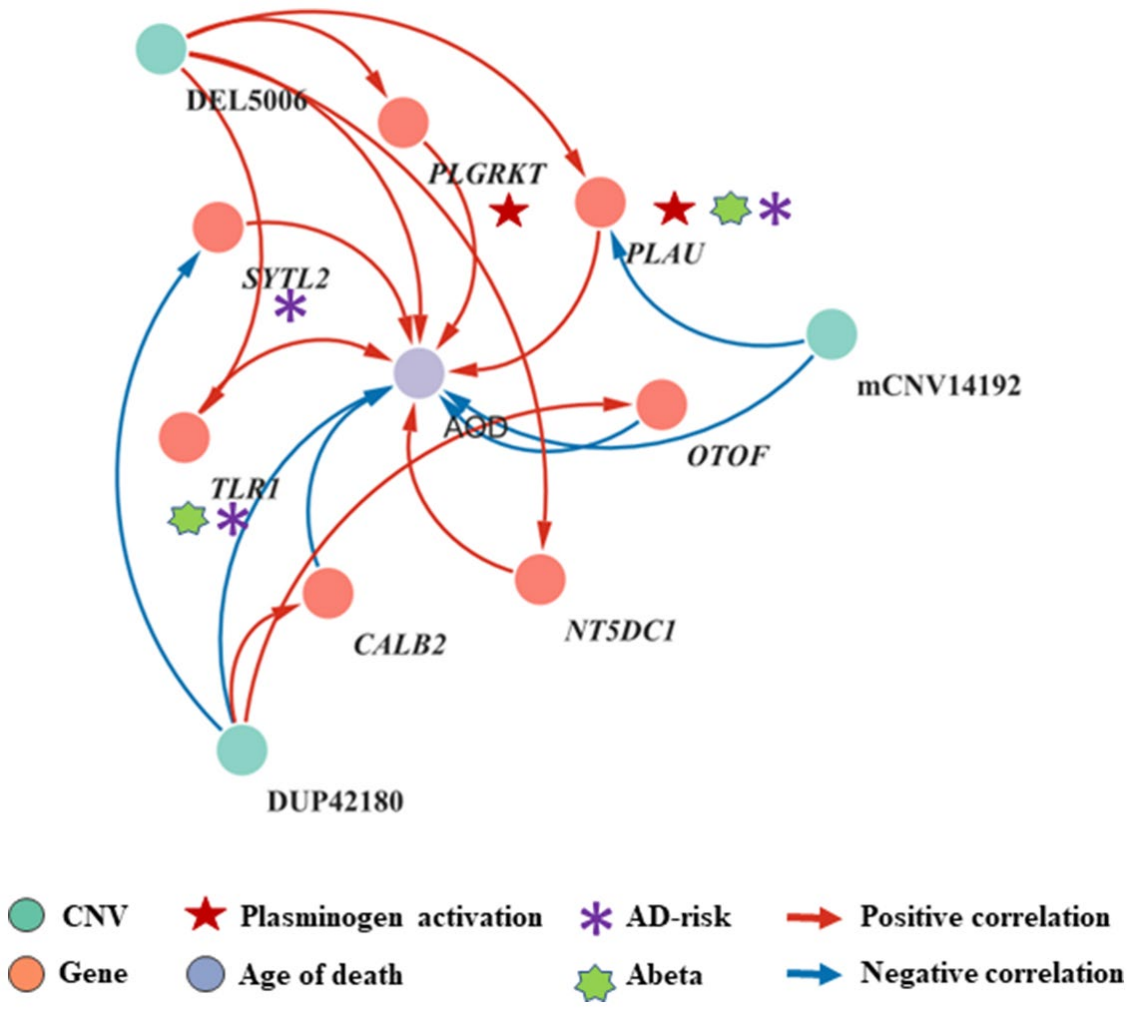
Causality network between AOD-correlated CNVs, AD longevity-causal genes, and AOD. The causality network connected 3 AOD-correlated CNVs, 7 AD longevity-causal genes, and AOD. Tangerine pink: genes, Turquoise blue: AOD-correlated CNVs; Lavender purple: AOD; Red arrows: positive correlation; Blue arrows: negative correlation; Red five-pointed star: plasminogen activation-related genes; Green seven-pointed star, Abeta genes; Purple asterisk, AD-risk genes.

To elucidate the inherent relationship between variations in gene expression levels and aging, we established a causality network that integrated AOD-correlated CNVs, presumptive AD-longevity-associated genes, and AOD by implementing the Causal Inference Test (CIT) (Millstein et al., 2016) (Methods 4.10). After excluding the aging caused variation in gene expression, there are three CNVs (i.e., DEL5006, mCNV14192, and DUP42180) regulating the expression level of seven genes (i.e., *PLGRKT*, *TLR1*, *PLAU*, *CALB2*, *SYTL2*, *OTOF*, and *NT5DC1*), and the expression level of these seven genes further regulates the age of death of AD patients, in the final causality network (Figure 5 and Table 2). We named the seven genes in the above causality network as AD longevity-causal genes. The copy number dosage of DEL5006 was positively correlated with most AD longevity positively causal genes (i.e., *PLGRKT*, *TLR1*, *PLAU* and *NT5DC1*), and served as a key longevity CNV loci. Meanwhile, the copy number dosage of the other two CNVs, which are DUP42180 and mCNV14192, were negatively correlated with AOD through regulating the expression of genes *OTOF*, *SYTL2*, *CALB2,* and *PLAU*. Interestingly, we further found two AD-risk genes, *PLAU* and *TLR1*, which functions in the Aβ clearance and degradation process (Ertekin-Taner et al., 2005; Liu et al., 2012) were positively correlated with AOD of AD patients. The copy number dosage of DEL5006 demonstrates a positive correlation with the expression level of *PLAU*. Conversely, the dosage of mCNV14192 shows a negative correlation with *PLAU* expression. The multi-CNV correlation pattern indicates the importance of *PLAU* in AD longevity. Notably, in the transgenic alpha murine urokinase-type plasminogen activator (αMUPA) mouse model, *PLAU* had been reported that its overexpression in the brain may extend mouse longevity by limiting food consumption (Miskin and Masos, 1997), which is consistent with our observation in AD patients (Figure 5).

**Table 2:** The summary of CNV-gene-AOD pairs in the causality network based on the physician’s overall cognitive diagnostic category at the time of death.

CNV	Gene	Trait	Beta (CNV-Gene)	P.adj (CNV-Gene)	Beta (CNV-AOD)	P.adj (CNV-AOD)	Cor. coefficient (Gene-AOD)	P.adj (Gene-AOD)	P.adj_cit
DEL5006	*PLGRKT*	AOD	1.25E+00	1.71E-02	1.00E+01	2.19E-02	1.03E-01	3.27E-02	3.07E-02
DUP42180	*OTOF*	AOD	1.15E+00	3.74E-02	−8.99E+00	1.58E-02	−1.65E-01	8.12E-04	3.85E-03
mCNV14192	*PLAU*	AOD	−1.52E+00	3.22E-03	−1.05E+01	3.25E-03	1.33E-01	6.32E-03	3.81E-03
DEL5006	*PLAU*	AOD	1.73E+00	4.28E-02	1.00E+01	2.19E-02	1.33E-01	6.32E-03	3.85E-03
DUP42180	*SYTL2*	AOD	−9.63E-01	1.96E-02	−8.99E+00	1.58E-02	1.04E-01	3.18E-02	2.72E-02
DUP42180	*CALB2*	AOD	1.77E+00	4.26E-02	−8.99E+00	1.58E-02	1.35E-01	8.94E-03	1.28E-02
DEL5006	*TLR1*	AOD	2.45E+00	2.67E-02	1.00E+01	2.19E-02	1.52E-01	5.45E-03	3.85E-03
mCNV14192	*TLR1*	AOD	−1.78E+00	2.04E-02	−1.05E+01	3.25E-03	1.35E-01	5.45E-03	3.85E-03
DEL5006	*NT5DC1*	AOD	9.95E-01	4.89E-02	1.00E+01	2.19E-02	1.52E-01	1.82E-03	3.85E-03
DEL5006	*PLGRKT*	AOD	1.25E+00	1.71E-02	1.00E+01	2.19E-02	1.03E-01	3.27E-02	3.07E-02

Beta (CNV-Gene), estimated effect size of CNV on gene expression; Beta (CNV-AOD), estimated effect size of CNV on age of death. P.adj, adjusted *p*-value calculated based on linear regression by Matrix eQTL and adjusted by FDR. P.adj_cit, the adjusted *p*-value from the Causal Inference Test with FDR correction. P.adj_cit < 0.05.

**Figure 6:**
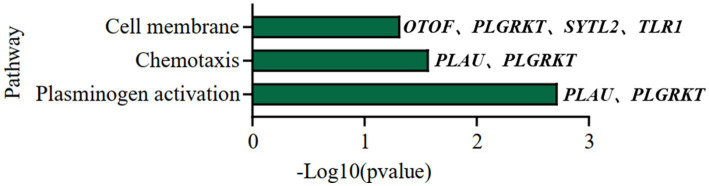
Pathway enrichment analysis of AD longevity-causal genes. The pathways of AD longevity-correlated genes obtained from DAVID. *p*-value <0.05, Fisher’s Exact test.

**Figure 7:**
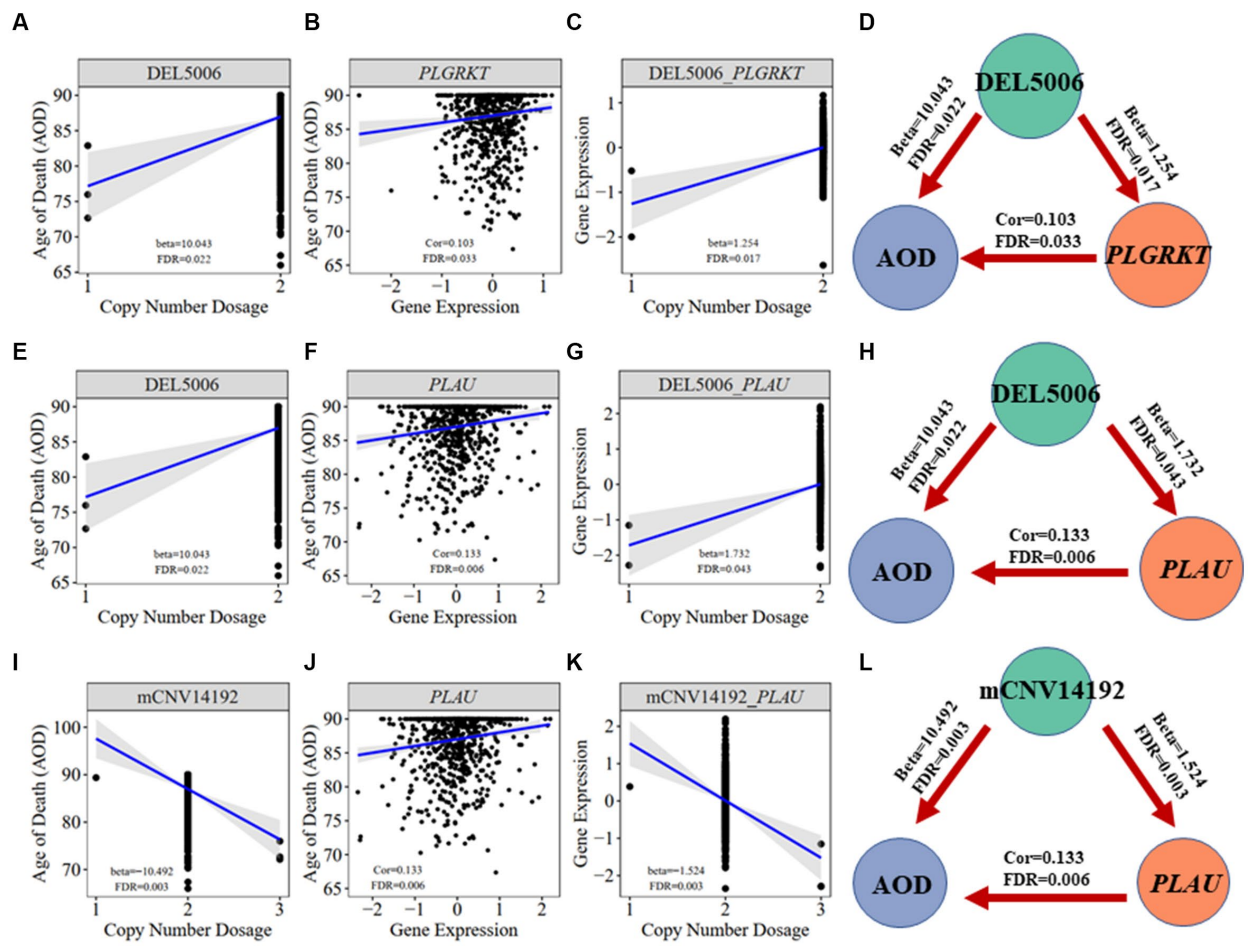
Correlation visualization of DEL5006, mCNV14192, *PLGKRT*, *PLAU*, and AOD. **(A)** Correlation between copy number dosage of DEL5006 and AOD. **(B)** Correlation between *PLGRKT* gene expression and AOD. **(C)** Correlation between copy number dosage of DEL5006 and *PLGRKT* expression. **(D)** Causality of DEL5006, *PLGRKT*, and AOD. **(E)** Correlation between copy number dosage of DEL5006 and AOD. **(F)** Correlation between *PLAU* gene expression and AOD. **(G)** Correlation between copy number dosage of DEL5006 and *PLAU* expression. **(H)** Causality of DEL5006, *PLAU*, and AOD. **(I)** Correlation between copy number dosage of mCNV14192 and AOD. **(J)** Correlation between *PLAU* gene expression and AOD. **(K)** Correlation between copy number dosage of mCNV14192 and *PLAU* expression. **(L)** Causality of mCNV14192, *PLAU*, and AOD. Beta: estimated effect size of CNV on AOD and gene expression; Cor: spearman coefficient; False Discovery Rate (FDR) < 0.05.

We further did the functional enrichment analysis for the seven AD longevity-causal genes by using DAVID database (Dennis et al., 2003) (Figure 6, Methods 4.11). The AD longevity-causal genes were significantly enriched in the plasminogen activation (Enrichment score = 843.9, *p* = 1.89E-3, FDR = 1.89E-2) and chemotaxis pathways, which involved two genes (*PLAU* and *PLGRKT*). *PLAU* encodes the urokinase-type plasminogen activator (uPA) controlling the key step in the plasminogen activation system (PAS) to convert the plasminogen to plasmin (Mahmood et al., 2018). Extensively studies had shown many human diseases controlled by dysfunctional PAS (Fay et al., 2007; Buckley et al., 2019; Baker and Strickland, 2020; Kumar et al., 2022). As a plasminogen receptor, *PLGRKT* is a major regulator to promote cell surface plasminogen activation and is highly colocalized with *PLAU* (Andronicos et al., 2010). Noted that the expression level of both *PLGRKT* and *PLAU* are positively correlated to AOD and regulated by the copy number dosage of DEL5006 (Figure 7), which further indicates the importance of plasminogen activation in longevity. Differential expression analysis showed *PLGRKT* and *PLAU* had no difference between the AD and normal groups indicating that *PLGRKT* and *PLAU*’s contribution to longevity was independent of AD status (Supplementary Table S8).

## 3. Discussion

The role of CNVs in influencing longevity has been confirmed in various human populations as demonstrated by prior studies (Kuningas et al., 2011; Nygaard et al., 2016; Zhao et al., 2018). Existing research also has shed light on the mechanisms by which genes contribute to longevity (Conneely et al., 2012; Melzer et al., 2020). Despite these advances, the specific genetic mechanism underpinning longevity of Alzheimer’s disease patients remains relatively unexplored.

In this study, we have taken a comprehensive approach by integrating CNV profiles, transcriptomic profiles, and meta-information of AD patients based on the ROSMAP cohort. Our construction of a CNV-gene-Age of Death causality network has yielded significant insights. We have successfully identified three critical CNVs - namely, DEL5006, mCNV14192, and DUP42180 - along with seven causal genes correlated with AD longevity. These genes include *PLGRKT*, *TLR1*, *PLAU*, *CALB2*, *SYTL2*, *OTOF*, and *NT5DC1*. The impact of these CNVs and genes on the longevity of AD patients appears to be independent of the severity of their AD status. This discovery provides new perspectives on the genetic influence on longevity in AD patients and opens up fresh avenues for potential therapeutic interventions. Further studies will be beneficial to validate these findings and uncover the precise mechanisms by which these CNVs and genes influence longevity in AD patients.

The role of the plasminogen activation system (PAS), implicated by the identification of the gene *PLAU* and *PLGRKT* in our study, deserves attention. Plasminogen activation has been related to numerous biological processes including tissue remodeling, wound healing, inflammation, fibrinolysis, extracellular migration, cell signaling, cellular migration and degradation of thrombosis within vascular (Castellino and Ploplis, 2005; Baker and Strickland, 2020). It is worth noting that the PAS has been implicated in the pathology of AD and the accumulation of fibrin triggers vascular or Aβ related pathology in AD patients (Petersen et al., 2018). Besides, the detrimental effects of Aβ on synapses are reversed by uPA and this protective effect works in the early stages of the onset of AD and is independent of the plasmin-induced cleavage of Aβ-containing plaques (Yepes, 2021). However, in our analysis, by excluding the bias associated with AD, our finding indicates PAS affects the longevity of AD patients not the pathology degree of AD.

**Figure 8:**
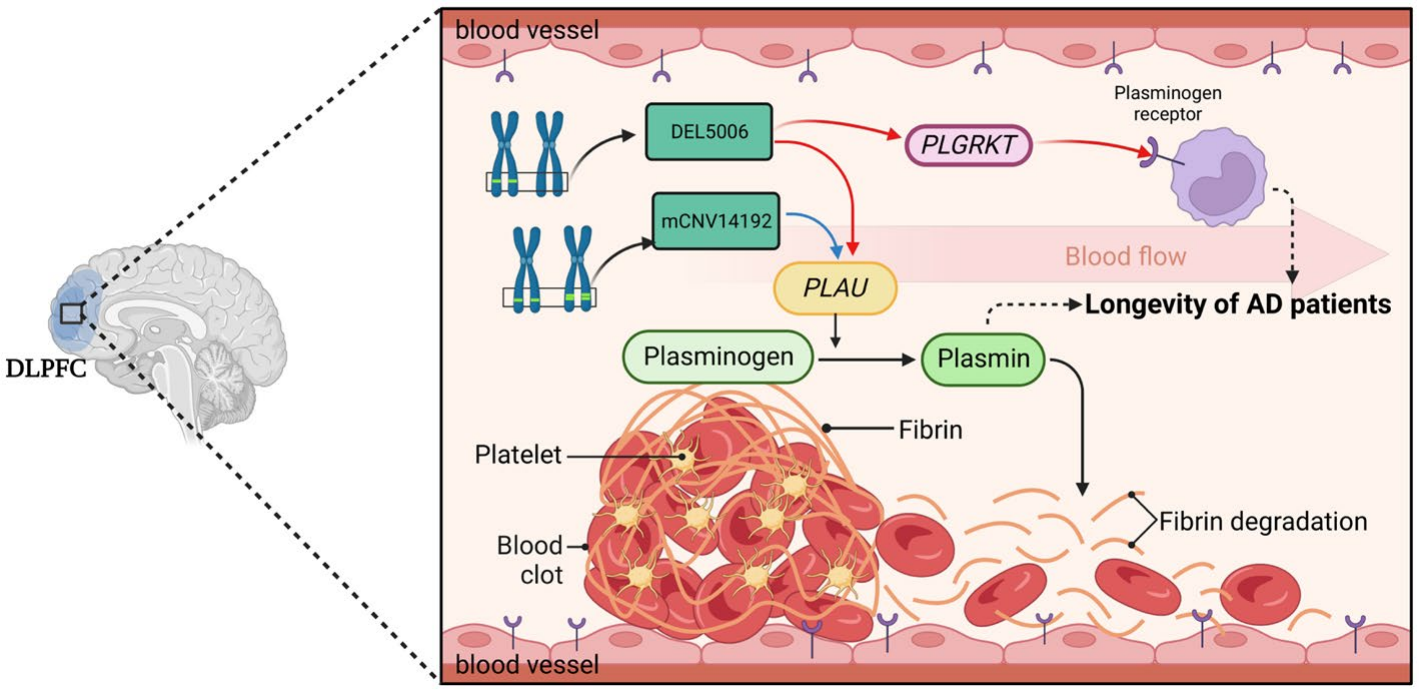
The hypothesis of DEL5006-*PLAU*/*PLGRKT*-AOD. The deletion at the DEL5006 loci resulted in the downregulation of *PLGRKT* encoding the plasminogen receptor and *PLAU* encoding the urokinase-type plasminogen activator (uPA), while the duplication at mCNV14192 loci also downregulated *PLAU* expression which caused the inhibition of the conversion from plasminogen to plasmin. This inhibition, in turn, led to the accumulation of fibrin in the blood vessel and impaired the longevity of AD patients. The picture was created by BioRender.

Our study has led us to propose several intriguing potential hypotheses for further exploration. Accumulation of thrombosis makes elders prone to stroke (Kalaria, 2000) and may have profound implications for their longevity. The first hypothesis we propose is that CNVs regulate PAS and finally affect cerebrovascular fibrinolysis. The deletion at the DEL5006 loci leads to the downregulation of *PLGRKT* and *PLAU* expression, while the copy number increase at the mCNV14192 loci down-regulates the *PLAU* expression. This downregulation may result in a reduction in plasminogen and plasmin levels, subsequently impairing fibrin degradation within the cerebrovascular system. Such a process could precipitate recurring thrombosis in the brains of Alzheimer’s patients. Consequently, this mechanism may contribute to reduced longevity in DEL5006 carriers (Figure 8). Our second hypothesis posits that CNVs modulate the plasmin-independent protective mechanism mediated by uPA-uPAR interaction. It has been observed that uPA treatment can augment the synaptic expression of neuronal cadherin (NCAD) via a uPAR-mediated, plasmin-independent mechanism. Moreover, the formation of NCAD dimers, induced by uPA, has been shown to confer protection to synapses against the harmful influence of soluble Aβ oligomers, as demonstrated in the 5xFAD mouse model (Diaz et al., 2020). Another study observed that pharmacologic inhibition or genetic deficiency of plasminogen activator inhibitor-1 (PAI-1) was protective against senescence in mouse model (Eren et al., 2014), which further supports our hypothesis of plasminogen activation and longevity. But the exact way the identified CNVs regulate the expression of target genes and how target genes affect the longevity of AD patients remains elusive. Further research is required to understand how these CNVs exert their influence and contribute to AD patient longevity. Moving forward, future work should involve rigorous experimental validation of these hypotheses. In-depth molecular and cellular studies could provide insight into the precise mechanisms underlying these associations.

Our study has several notable limitations. Firstly, the findings, while statistically significant and derived from rigorous analytical methods, lack experimental validations. These findings warrant further verification through experimental studies. Secondly, the identified CNVs and genes’ functional implications, and their interactions, necessitate further scrutiny. An additional constraint is that gene expression was measured solely in the DLPFC region. Consequently, it remains unclear whether the identified causality network is applicable to other brain regions—an aspect which merits further exploration. Furthermore, the population frequency of the AOD-correlated CNVs we identified is low. The correlation coefficient between the genes and AOD is also quite small, implying that there are likely a plethora of other genetic risk factors influencing the longevity of AD patients, which are beyond the scope of the candidate CNVs and genes identified in this study. Another limitation is the lack of drug records. There are some antiaging drugs used in clinical application, for example, Metformin (Barzilai et al., 2016). Metformin is one of the medications for Type 2 Diabetes Mellitus (T2DM). However, a growing number of studies have confirmed the positive correlation between T2DM and AD (Schilling, 2016; Chung and Lee, 2021). The previous study showed that metformin exhibits rapid penetration of the blood–brain barrier and subsequent accumulation within various brain regions (Łabuzek et al., 2010), enabling its impact on the central nervous system in rat model (El-Mir et al., 2008). Metformin could ameliorate AD symptoms by inhibiting neuronal loss, activating atypical PKC-CREB-binding protein (aPKC-CBP) signaling pathway in neural precursors, or decreasing the accumulation of Aβ (Liao et al., 2022). These antiaging drugs are potential covariates needed to consider. Unfortunately, because the ROSMAP cohort did not provide a medication history, we did not consider the drugs used in our current analysis. Human longevity is a complex phenotype, affected by a multitude of genes and environmental factors. Thus, we must interpret our findings within the context of this complexity, acknowledging the likely existence of a myriad of other genetic and environmental contributors to Alzheimer’s patient longevity (Figure 8).

In conclusion, while we have identified key genetic elements that might influence AD patient longevity, we acknowledge that the intricacies of these relationships are likely to be far more complex. Our study provides a steppingstone and offers promising directions for future research in the genetic mechanisms influencing longevity in AD patients.

## 4. Methods

### 4.1. CNV profiles based on the whole-genome sequencing data from the ROSMAP AD cohort

The CNV profiles of the 1,127 individuals from the ROSMAP cohort were downloaded from the AD Knowledge Portal^1^ and the CNV calling details were described in previous study (Ming et al., 2022). Here was the main procedure: (1) CNV calling at an individual level for each sample through CNVnator and Pindel. The CNV calling results were integrated by MetaSV and we got the first dataset of individual-level CNVs. (2) Use Delly2 to generate the second set of individual-level CNVs. (3) Got the final individual-level CNVs set by merging the first and second CNVs set. (4) The generation of CNVs at population level. If the reciprocal overlapping region of CNVs from different individuals was higher than 50%, the CNVs were merged into one CNV. (5). Due to the merging of the CNVs, the CNV boundaries could change, and copy number dosage of each sample was re-genotyped using the genotype function of CNVnator.

### 4.2. Transcriptomic data

The transcriptomics data was downloaded from the AMP-AD portal (synapse ID: syn3388564) and processed in details in previous study (Mostafavi et al., 2018). We further adjusted the gene expression value by excluding the bias of disease status with function lmFit in R package limma (Ritchie et al., 2015). We treated AD pathological traits, which are cogdx, braaksc, and ceradsc, as covariate independently to exclude the bias from the severity of AD status. Finally, we used residual obtained from the linear regression to represent the gene expression caused by AOD. The residual values were calculated using the following formula:


(1) Residual=g−α×cogdx

“g” is gene expression value, α is coefficient values obtained from lmFit function in limma, while cogdx represents the physician’s overall cognitive diagnostic category at the time of death in the ROSMAP cohort. All afterward analysis was performed using the adjusted gene expression value.

The differential gene expression (DEG) analysis was performed between AD and Normal group using limma. The cut-off was setted as FDR < 0.05 and |logFC| >1.

### 4.3. Clinical and pathological trait data

The clinical meta information was downloaded from the AMP-AD portal (Synapse ID: syn3157322). We used the information including age at death, race, sex, braak, ceradsc, and cogdx score. In the afterward analysis, to exclude population bias, we only considered individuals who were North American whites. All individuals were classified into two groups based on their final Clinical Consensus Diagnosis (cogdx): 762 individuals with Alzheimer’s disease (AD; scores of 2–5) and 365 individuals with normal cognition (NL; score of 1). To facilitate our analysis, we re-coded the ceradsc score as follows: (1) for Normal, (2) for probable, (3) for possible, and (4) for definite AD.

### 4.4. Workflow of this study

In this research, we integrated WGS and transcriptomics data to explore the potential mechanism of longevity of AD patients. As depicted in Figure 2, we primarily utilized Matrix eQTL to identify AOD-correlated CNVs and genes, and subsequently identified presumptive aging causal genes that were correlated with both AOD-correlated CNVs and AOD through Spearman correlation analysis. Next, we integrated the presumptive aging causal genes and AOD information into cit R package (Millstein et al., 2016) to construct a causality network, which we analyzed to reveal the underlying mechanisms of aging.

### 4.5. Correlation analysis of copy number dosage and age of death

We used the function Matrix_eQTL_engine with a linear regression model in the R package Matrix eQTL (version 2.3) (Shabalin, 2012) for the correlation analysis. We tested the correlation between CNV dosage and AOD based on linear regression model, as the formula:


(2) AOD=β×CNV+α×SEX+γ×DiseaseStatus+ε

The age of death which was higher than 90 was replaced with 90 in the meta-information file of ROSMAP. The subjects with 0 standard deviations of CNVs were excluded to make sure the CNV dosage in the whole subjects was different. We used pseudo-code for sex (Female = 0, Male = 1). There were three AD pathological traits, cogdx, braaksc, and ceradsc which were adjusted as well as sex in the linear regression processing and we detected 12, 12, and 11 CNVs correlated with AOD, respectively, (FDR < 0.05). Finally, we only considered the 10 consensus AOD-correlated CNVs for all three traits.

### 4.6. AOD-correlated CNVs frequency

To determine the frequency of AOD-correlated CNVs in different groups of the ROSMAP cohort, we calculated the ratio of individuals with AOD-correlated CNVs among the whole cohort, only AD group, and only Normal groups. We further performed a Chi-Squared Test to compare the CNV frequency between AD and Normal groups.

### 4.7. Correlation analysis of CNV dosage and gene expression level

We used individuals with both WGS and transcriptomics data in the correlation analysis of CNV dosage and gene expression level to establish a robust correlation between the identified genes and AOD-CNVs in the context of the same individuals’ genetic and transcriptional profiles.

Like the CNV-AOD correlation analysis, Matrix eQTL was also used for the correlation analysis between AOD-correlated CNVs and gene expression. We performed a linear additive model to adjust sex and disease status and got 110 consensus genes whose expression significantly correlated with 10 AOD-correlated CNVs and formed 125 CNV-Gene pairs.

### 4.8. Correlation analysis for the age of death and gene expression

A linear model was applied to exclude bias from disease status of transcriptomics data by using the lmFit function of the R package limma (Ritchie et al., 2015). Spearman correction was calculated between log2-transformed expression values output from limma and AOD. Based on cogdx, braaksc, and ceradsc, there were 1,294, 2056, and 2,774 AOD-correlated genes were identified, respectively, under the significance level of FDR < 0.05.

### 4.9. Construct CNV-gene-AOD correlation network

By intersecting 1,294, 2056, 2,774 AOD-correlated genes and 110 genes correlated to AOD-correlated CNVs, we finally focus on 14, 17, and 21 genes which were both correlated with AOD and AOD-correlated CNVs while using braaksc, cogdx, and ceradsc. We used the 17 genes under cogdx forming 23 CNV-Gene-AOD pairs to construct CNV-Gene-AOD correlation network. The correlation pairs were defined as the edges to link their responding nodes. Then Cytoscape (3.9.1) (Shannon et al., 2003) was used for network visualization.

### 4.10. Construct CNV-gene-AOD causality network

The CNV-gene-AOD causality network was built by combining significantly correlated CNV-AOD, CNV-gene, and gene-AOD pairs from the ROSMAP cohort. We compared the probability of two scenarios:

Scenario 1: CNV-Gene-AOD, indicating that CNV contributed to AOD via gene expression change.

Scenario 2: CNV-AOD-Gene, indicating that aging causes the variation of gene expression.

To estimate the likelihood of the two scenarios, we utilized the Causal Inference Test (CIT) (Millstein et al., 2016). We choose the scenario with more significant FDR as the more likely scenario. Eventually, we identified three AOD-correlated CNVs that regulated seven genes and affected AOD, which were used to construct the causality network. The correlation pairs between these genes and CNVs were defined as edges that linked their corresponding nodes in the network. We utilized Cytoscape (version 3.9.1) to visualize the network. In the network diagram, turquoise blue, tangerine pink, and lavender purple circles represent CNVs, genes, and age of death, respectively. The red star symbol indicates the gene that was enriched for the plasminogen activation pathway, while the purple asterisk represents the AD-risk genes. The red and blue edges represent positive and negative correlations, respectively. In the causality network, arrows are used to indicate the direction of causality.

### 4.11. Functional enrichment

Performed pathways enrichment analysis on seven AD longevity-causal genes using DAVID, with Fisher’s Exact test, as the following equation:


(3) $$P=\frac{\left(\begin{array}{c} a+b\\ a \end{array}\right)\left(\begin{array}{c} c+d\\ c \end{array}\right)}{\left(\begin{array}{c} n\\ a+c \end{array}\right)}$$

“a” is the genes we input that are mapped to the pathway, “b” is the genes mapped to the pathway based on the whole genome level, “c” is the gene we input that are not mapped to the pathway, “d” is the genes that are not mapped to the pathway on the whole genome level, and “n” represent the total number of genes in the genome (i.e., *n* = a + b + c + d).

The background was defined as the 18,364 genes detected in the transcriptomics data.

### 4.12. Survival analysis

Conducted survival analysis for 10 AOD-correlated CNVs using AOD of ROSMAP cohort via the survfit function in R package survival (Therneau, 2023). All individuals were classified into separate groups based on the copy number dosage of each AOD-correlated CNV. All individuals exhibited death events responding “event = 2” in the survfit function. Log-rank test was used to compare the survival curves between individuals with different copy number dosage.

## Data availability statement

The original contributions presented in the study are included in the article/Supplementary material, further inquiries can be directed to the corresponding author.

## Ethics statement

Ethical approval was not required for the studies involving humans because Ethical approval was not sought for this study, in compliance with local laws and institutional guidelines. The research presented in this paper is based solely on publicly available datasets, which contain no personally identifiable information or sensitive data. The datasets were collected and made public in a manner that is in accordance with all applicable ethical standards, and this study did not involve any interaction with human or animal subjects. The studies were conducted in accordance with the local legislation and institutional requirements. The human samples used in this study were acquired from gifted from another research group. Written informed consent to participate in this study was not required from the participants or the participants’ legal guardians/next of kin in accordance with the national legislation and the institutional requirements.

## Author contributions

CM designed the project. YH did the bioinformatics analysis. CL processed the raw WGS, transcriptomic data, and provided complementary analyses. YH and CM wrote and edited the manuscript. HW worked on response to comments from reviewers and involves comparison different public databases, such as DGV, 1000 Genome Project, and GnomAD. All authors reviewed and edited the paper. All authors have read and approved of the paper.

## Footnotes

^1^ http://doi.org/10.7303/syn26254632
