# Supplementary material for: Effects of copy number variations on longevity in late-onset Alzheimer’s disease patients: insights from a causality network analysis
Source: Front Aging Neurosci. 2023 Nov 2;15:1241412. doi: 10.3389/fnagi.2023.1241412 (PMC10652415; doi:10.3389/fnagi.2023.1241412)
Supplement: Supplementary file 2 [file Image_1.pdf]

Supplementary Figure

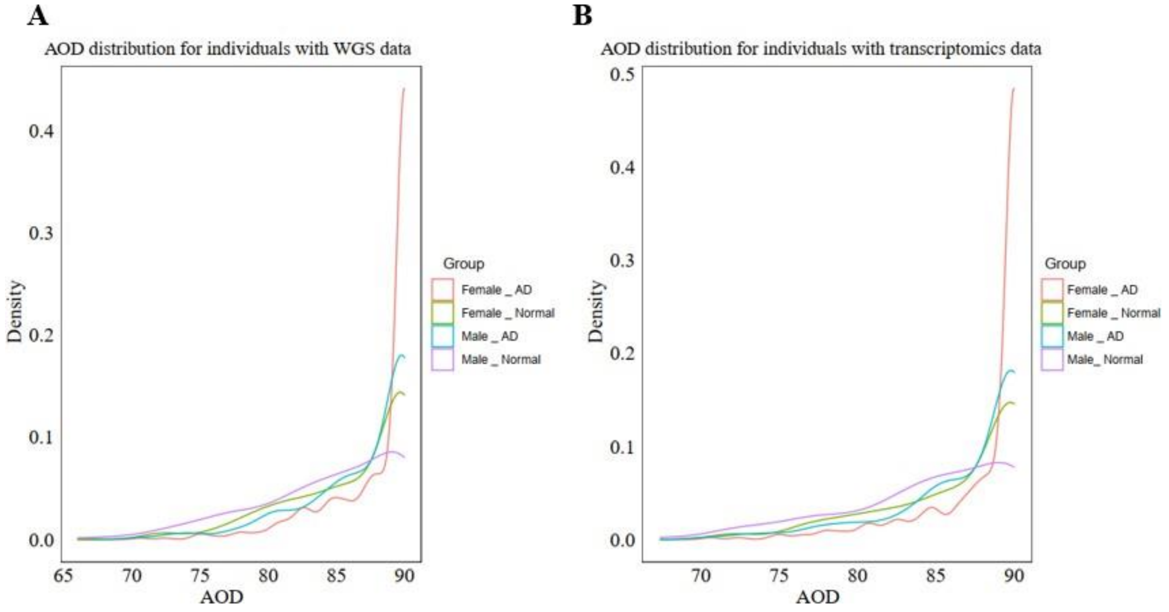

Figure S1. The distribution of AOD for different groups with WGS and transcriptomics data. The individuals were departed into Female_AD, Female_Normal, Male_AD, and Male_Normal groups.

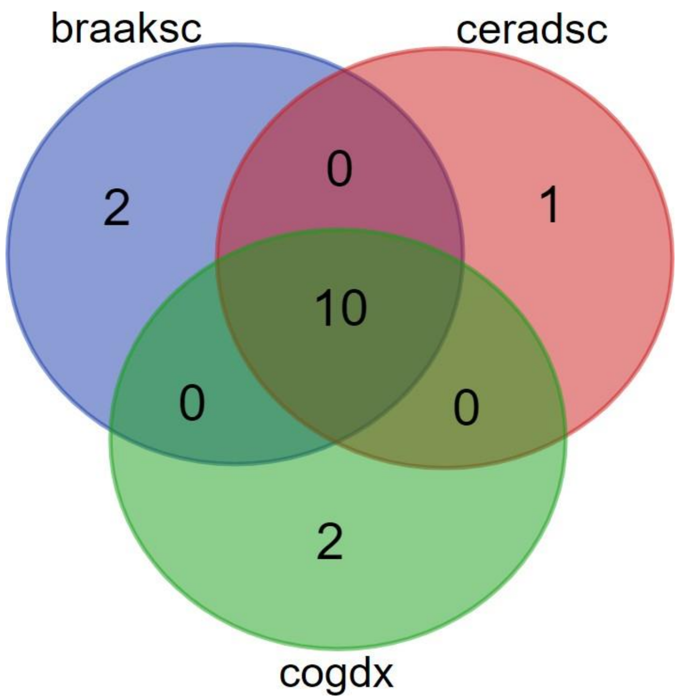

Figure S2. The Venn plot shows the intersection of AOD-correlated CNVs after adjusting different AD pathologic traits, i.e., braaksc, ceradsc and cogdx, independently.

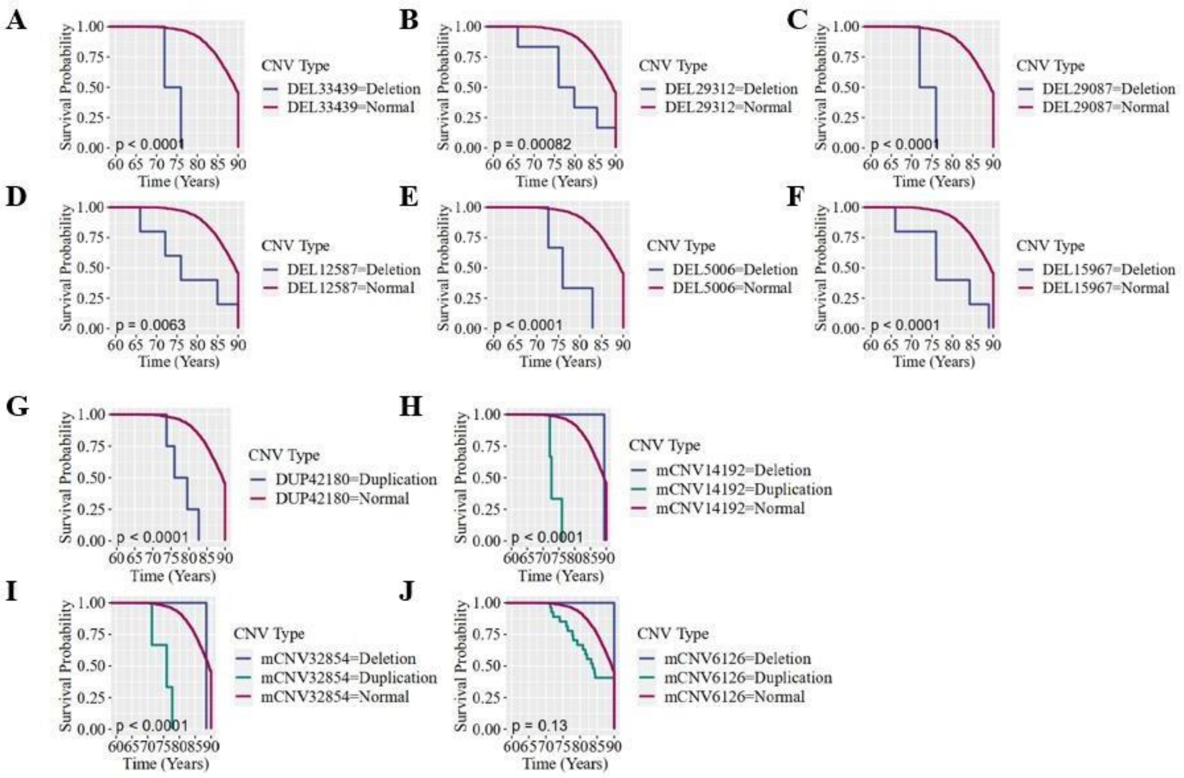

Figure S3. Survival of individuals with AOD-correlated CNVs. All individuals were classified into different groups based on copy number dosage. Normal: copy number dosage = 2; Deletion: copy number < 2; Duplication: copy number dosage > 2. p, log-rank test. $p < 0.05$

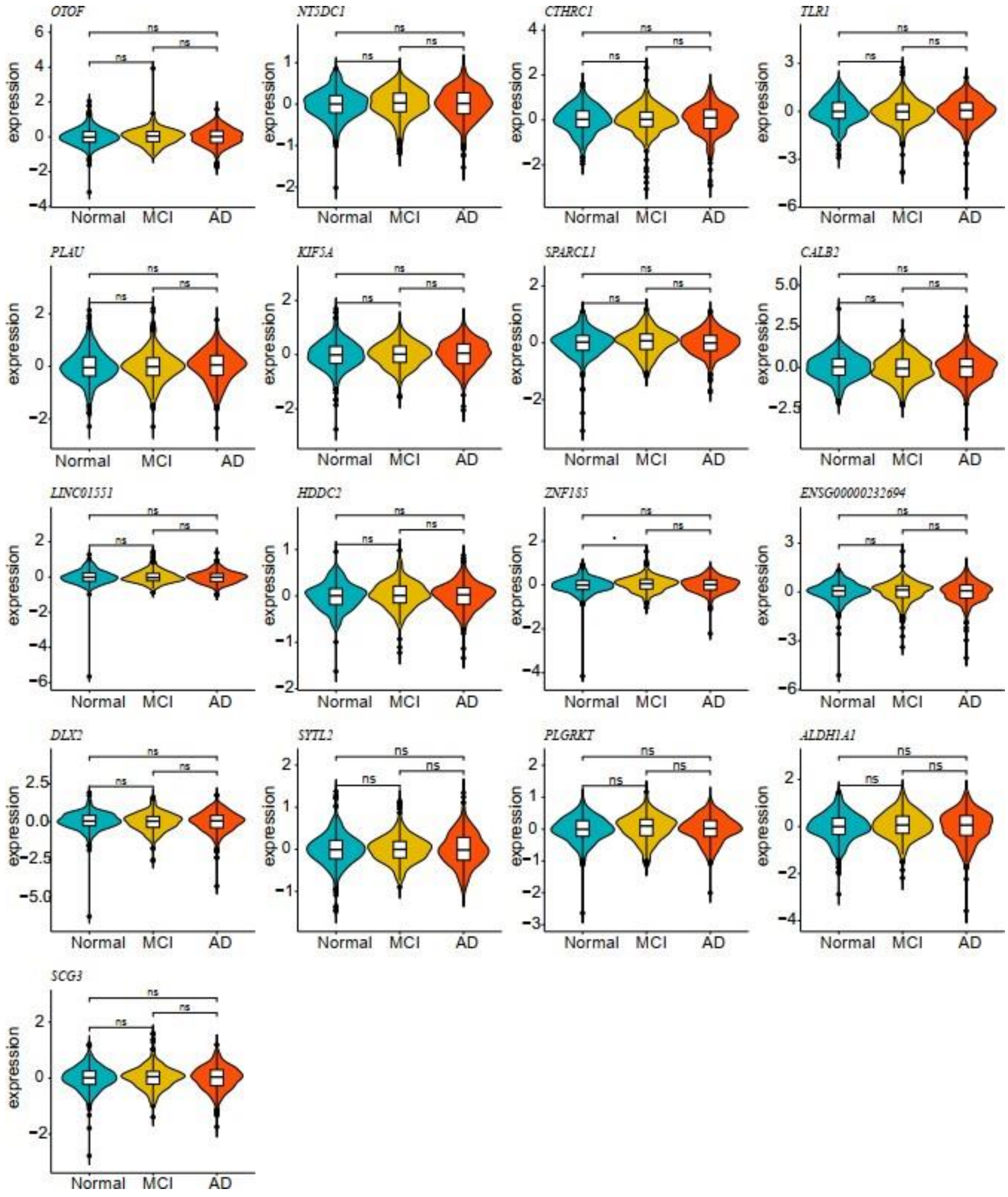

Figure S4. Gene expression of presumptive AD longevity-correlated genes in different groups. The violin plot shows the expression level of the presumptive AD longevity-correlated genes after adjusting disease status (cogdx). There is no significant disease status effect on the gene expression level after the co-variance adjustment. The gene expression level is comparable among groups with different disease status. The cogdx of the AD group is larger than 3, the cogdx of the Mild Cognitive Impairment (MCI) group is 2 or 3, and the cogdx of the Normal group is 1.
